# Complete genome sequence of bacteriophage VvAW1, which infects *Vibrio vulnificus*

**DOI:** 10.4056/sigs.2846206

**Published:** 2012-07-20

**Authors:** Olivia D. Nigro, Alexander I. Culley, Grieg F. Steward

**Affiliations:** University of Hawaii Department of Oceanography Center for Microbial Oceanography: Research and Education Honolulu, HI

**Keywords:** Bacteriophage, vibriophage, *Vibrio vulnificus*, podophage, aquatic, virus

## Abstract

Investigating the bacteriophages of vibrios has led to significant insights into the evolution and pathogenicity of their host strains. This report presents the first complete genome sequence of a bacteriophage that infects the deadly human pathogen *Vibrio vulnificus.* The phage was isolated from the surface waters of the Ala Wai Canal, which is part of an urban watershed in eastern Honolulu, Hawaii, USA. The phage particle is icosahedral, with a diameter of 35-40 nm, and a small non-contractile tail. The genome was sequenced in its entirety, rendering a 38 kb sequence located on a single, linear, circularly permuted chromosome. Here, we present the annotation and genomic features of the bacteriophage, VvAW1.

## Introduction

*Vibrio vulnificus* is an opportunistic, but formidable pathogen that can cause lethal infections in humans [1]. *V. vulnificus* is responsible for upwards of 95% of shellfish-related deaths in the US, and is a common cause of lethal infections resulting from exposure of wounds to marine and brackish waters. Outbreaks of vibrio infections are often associated with flooding caused by natural disasters [2]. Bacteriophages play important, but sometimes competing, roles in the epidemiology of many bacterial pathogens, including vibrios [3,4]. A temperate phage enhances the virulence of *V. harveyi*, for example [5]. In the case of disease cholera the temperate phage, CTXØ, contributes to the virulence of the causative agent, *V. cholerae*, via lysogenic conversion [6], but other lytic bacteriophages appear to ameliorate cholera epidemics by lysing the pathogen [7]. Currently, little is known about phages that infect *V. vulnificus*, but they are reported to be diverse and abundant in the Pacific Northwest and Gulf Coast regions of the US [8]. *V. vulnificus* phages could be a major contributor to the observed variability in virulence among host strains through the processes of horizontal gene transfer and lysogenic conversion. As a first step toward evaluating these possibilities, we are undertaking the isolation and detailed characterization of model phage-host systems. We chose the Ala Wai Canal in Honolulu, HI as our sampling site, since the waters of the canal appear to have been the source of a lethal infection by *Vibrio vulnificus* [9]. In 2009, we isolated a bacteriophage that infects *Vibrio vulnificus* from the canal. Here we present a description and annotation for the complete genome sequence of Vibrio phge VvAw1.

## Classification and Features

Both the Vibrio phage VvAW1, and its host *V. vulnificus* strain V93D1V were isolated from the Ala Wai Canal, a 3.1 km long, man-made waterway located on the southern coast of Oahu that separates Waikiki and urban Honolulu [10]. A watershed covering 42.4 km^2^ drains into the Ala Wai Canal, and the streams feeding the canal drain through urban areas of Honolulu, resulting in anthropogenic contamination [11]. The influx of fresh water from the streams, and the influx of seawater through the Ala Wai Harbor, creates a salinity gradient with a typical salt-wedge structure [10]. The salinity in the canal ranges from 0 to 35 ‰, depending on precipitation and tidal fluctuations (Nigro and Steward, unpublished data). Because of the local tropical climate, the temperature range of the canal is relatively narrow, usually ranging from 20 to 30 °C (Nigro and Steward, unpublished data). The host *V. vulnificus* strain, V93D1V, is a 16S rRNA “Type A”, which is not commonly associated with pathogenic infection [12].

**Figure 1:**
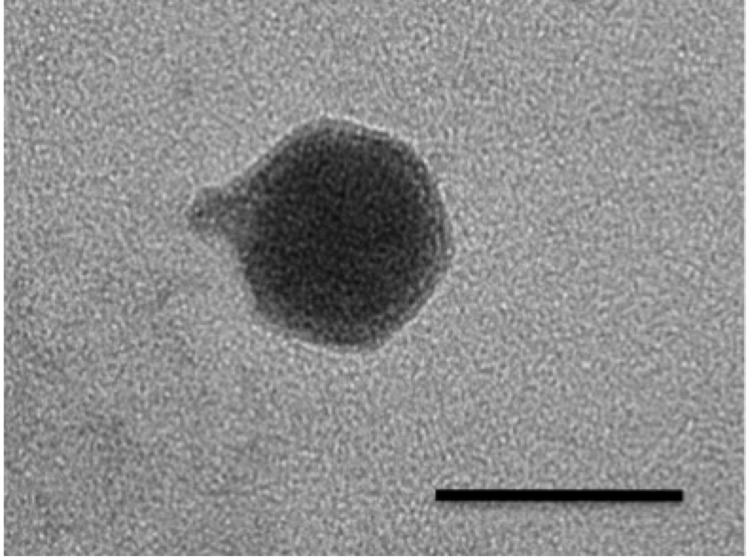
Transmission electron micrograph of a vibriophage VvAW1 particle. Scale bar equals 50 nm.

**Figure 2:**
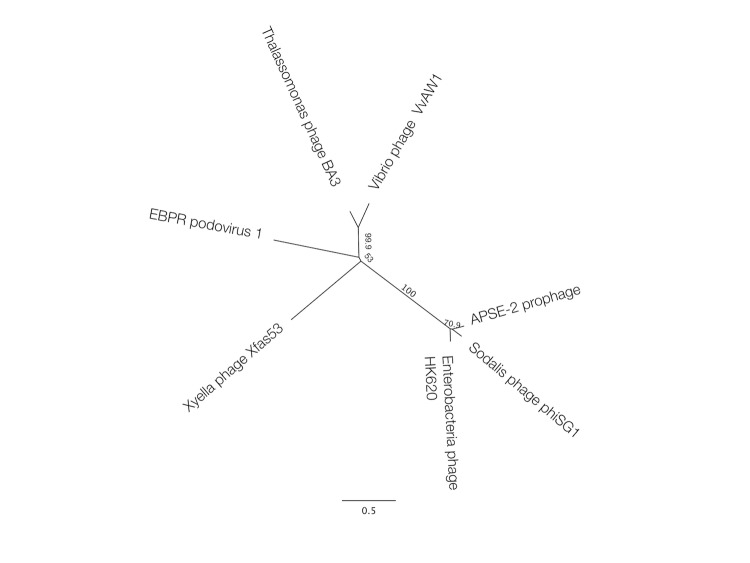
Unrooted maximum likelihood tree highlighting the position of Vibriophage VvAW1 relative to its most similar homologs by BLAST search (E-value > 10e-5). All of the phages in the tree are classified as podoviruses, with the exception of phiSG1 and APSE-2 prophage, which are unclassified. The phages and the corresponding GenBank accession numbers for their capsid protein genes are: EBPR podovirus AEI70875; Xyella phage Xfas53, YP_003344924; Thalassomonas phage Ba3, YP_001552282; Sodalis phage phSG1, YP_516191; APSE-2 prophage, YP_002924439; and Enterobacteria phage HK620, NP_112079. These sequences were aligned using the MAFFT alignment program, version 6.814b, using auto settings; and the maximum likelihood tree was built using PHYML with a Whelan and Goldman substitution model, and was bootstrapped 10,000 times using the Geneious software package v 5.5.7 [14].

The phage particle is icosahedral, with a capsid diameter of 43-45 nm and a tail-plus-capsid diameter of 52 nm (Figure 1 and Table 1). The tail appears to be short and non-contractile, which is commonly seen in members of the *Podoviridae* family [13]. The genome of the phage is made up of double-stranded DNA, and appeared by pulsed-field gel electrophoresis to be around 40kb in length (data not shown). These structural observations, combined with phylogenetic evidence (Figure 2) have resulted in the tentative classification of VvAW1 as a member of the *Podoviridae* family, with an unassigned genus.

**Table 1:** Classification and general features of Vibro phage VvAW1

**MIGS ID**	**Property**	**Term**	**Evidence code**^a^
	Current classification	Superkingdom: Viruses, dsDNA viruses, no RNA phase	IDA
		Order *Caudovirales*	
		Family *Podoviridae*	
		Genus unassigned	
		Species unassigned	
			
	Virion shape	Icosahedral	IDA
			
			
			
			
			
			
			
MIGS-6	Habitat	Aquatic	IDA
MIGS-6.3	Salinity		
MIGS-22	Oxygen		
MIGS-15	Biotic relationship	Obligate intracellular parasite of *V. vulnificus*	IDA
MIGS-14	Pathogenicity	Infective phage of *V. vulnificus*	IDA
MIGS-4	Geographic location	Ala Wai Canal, Honolulu, HI, USA	IDA
MIGS-5	Sample collection time	4/29/09, 08:40 AM	IDA
MIGS-4.1	Latitude	21.275510	IDA
MIGS-4.2	Longitude	-157.817869	IDA
MIGS-4.3	Depth	Surface	IDA
MIGS-4.4	Altitude		

a) Evidence codes - IDA: Inferred from Direct Assay.

## Genome sequencing information

### Genome project history

Vibriophage VvAW1 is the first genome sequence publicly available for a phage infecting *V. vulnificus*. As such, this sequence is a significant contribution to limited data sets of vibriophage and podovirus genomes. This genome was selected for sequencing as a first step toward better understanding the roles of bacteriophages in the ecology and virulence of *V. vulnificus*. DNA sequencing and assembly was performed at the University of Hawai‘i at Mānoa, Department of Oceanography, Center for Microbial Oceanography: Research and Education (C-MORE) using the university’s Advanced Studies in Genomics, Proteomics and Bioinformatics (ASGPB) sequencing facility. Genome annotation was performed both in-house as well as by submission to the IMG-ER genome annotation pipeline (Department of Energy Joint Genome Institute) [15,16]. A summary of the project information is presented in Table 2.

**Table 2:** Project information

**MIGS ID**	**Property**	**Term**
MIGS-31	Finishing quality	Closed
MIGS-28	Libraries used	1 genomic library: 800-1200 kb
MIGS-29	Sequencing platforms	Sanger
MIGS-31.2	Fold coverage	8.73×
MIGS-30	Assemblers	Sequencher version 4.10.1 (Gene Codes Corporation)
MIGS-32	Gene calling method	GeneMark.hmm, RAST version 4.0, and IMG-ER using Prodigal
	Genome Database release	GenBank
	Genbank ID	JQ801337
	Genbank Date of Release	May 31, 2012
	GOLD ID	Gi14686
	Project relevance	Virulence, Limited Sequence

### Growth conditions and DNA isolation

Viral strain VvAW1 was isolated from waters collected at the head of the Ala Wai Canal in Honolulu, Hawai‘i. On April 4, 2009, a one-liter surface water sample was collected, transported and processed within two hours of collection. Concurrent measurements of temperature and salinity were made at the time of collection. The water sample was filtered through a 0.22 µm membrane filter (Sterivex; Millipore) using a peristaltic pump. The filtered Ala Wai water was then supplemented with six strains of *V. vulnificus* and incubated at room temperature overnight in order to increase the numbers of any *V. vulnificus* phages that were present through infectious replication. The water was then filtered again through a 0.22 um Sterivex filter. The presence of phages was checked by an agar overlay plaque assay against each of the six inoculated strains. Each strain was incubated separately with 0.05 ml of the virus-containing filtrate for 10 minutes. This mixture was combined with molten soft tryptic soy agar (TSA) agar (0.6% agar w/v) and poured onto a TSA plate. Plates were incubated at 37 °C for 72 hours, and examined for plaques every 24 hours. After 72 hours, one strain, V93DIV, had 8 plaques. A single plaque was harvested, serially diluted and plated using the soft-agar overlay technique serially, three times to ensure the isolation of a single strain of phage. Following isolation, a bacterial lawn of strain V93D1V was fully lysed to obtain a high virus titer.

Viral particles were purified in a continuous equilibrium buoyant density CsCl gradient, spun at 29,000 rpm for 92.5 hours at 20 °C in an XL-80K Ultracentrifuge (Beckman) with a SW-41 rotor. CsCl was exchanged with TE buffer (10 mM Tris, 1 mM EDTA, pH 8) by centrifugal ultrafiltration and total nucleic acids were extracted using silica-based spin columns (QIAGEN DNeasy Blood and Tissue Kit) according to the manufacturer’s instructions. Genomic DNA was hydraulically sheared to 1000-1200 bp-sized fragments (HydroShear; GeneMachines). Size-selected DNA was end-repaired (DNATerminator End Repair Kit, Lucigen) and ligated into a pSMART HC KAN vector (Lucigen) and then transformed via electroporation into DH5alpha *E. coli* 10G SUPREME electrocompetent cells (Lucigen). Colonies were picked (n=288) and grown in CircleGrow (MP Biomedicals) liquid media plus kanamycin and plasmid DNA was isolated.

### Genome sequencing and assembly

The genome was sequenced using Sanger sequencing. Sequencing was performed by the Advanced Studies of Genomics, Proteomics and Bioinformatics (ASGPB) Sequencing Center at the University of Hawai‘i at Mānoa (Honolulu, HI), using Applied Biosystems BigDye terminator chemistry and was run on an ABI 3730XL capillary-based DNA sequencer. The genome was assembled using Sequencher version 4.10.1 (Gene Codes Corporation). Initial reads were assembled and formed two contigs. PCR was used to close two gaps into a circular topology (in total, 6 sets of PCR primers), however restriction enzyme digestion indicated the genome of VvAW1 is linear, circularly permuted, and terminally redundant. PCR was also used to re-sequence areas with low coverage. Assembly was manually curated for errors. Final coverage of the genome is 8.7×.

### Genome annotation

Open reading frames (ORFs) were determined using a combination of three gene calling methods, i) the Genemark.hmm 2.0 gene prediction program [17], ii) the RAST (Rapid Annotation using Subsystem Technology) genome annotation server [18] and iii) the Integrated Microbial Genomes-Expert Review (IMG-ER) platform developed by the Joint Genome Institute, Walnut Creek, CA, USA [15]. ORFs that were identified by only one of the three methods and which showed no homology to known proteins (E-value < 1e^-5^) were not included in the annotation. The predicted ORFs were translated and used to search the National Center for Biotechnology Information (NCBI) non-redundant database, the Conserved Domain Database (CDD), TIGRFam, Pfam, SMART, PRK, COG, and InterPro databases. The tRNAScanSE tool [19] was used to find tRNA genes. Additional manual functional annotation was performed within the IMG platform [20], within the Artemis/ACT package [21,22] and using the Geneious software package v 5.5 [14]. The complete genome sequence was submitted to GenBank and assigned the accession number JQ801337.

## Genome properties

**Figure 3:**
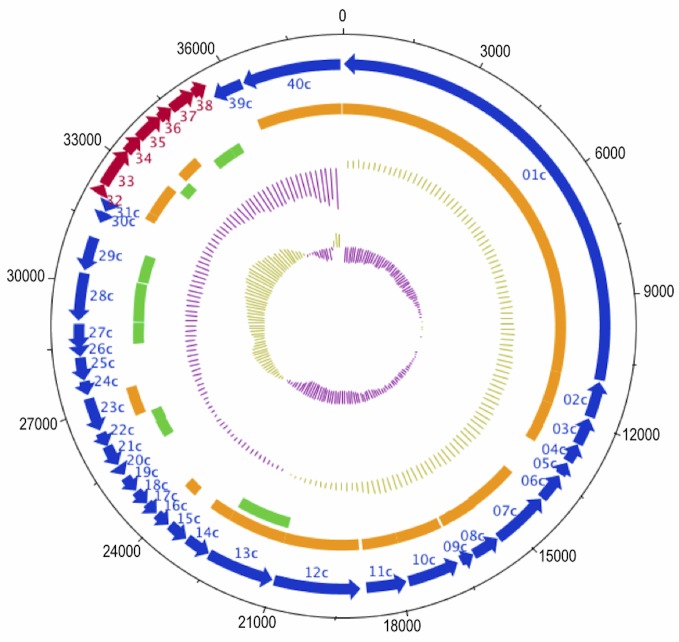
Genome map of VvAW1. The outer two tracks show numbered predicted genes and direction of transcription (red: forward direction, blue: reverse). The orange and green tracks indicate genes with significant sequence similarity (> 10e^-5^) to Thalassomonas phage Ba3, and Pseudomonas phage F116, respectively. Moving inward, the tracks show the %GC content (purple=low %GC) and GC skew ([G-C]/[G+C]) (innermost) of the genome.

The properties and the statistics of the genome are summarized in Tables 3-5.The genome has a total size of 38,682 bp of unique sequence, with one circularly permuted, terminally redundant, linear chromosome (49.1% GC content) (Figure 3). A total of 40 genes were predicted, all of which are protein-coding genes. Of these predicted protein-coding genes, eight were assigned to a putative function three were assigned to conserved, but unknown functional categories, and the remaining were annotated as hypothetical proteins. Although only eight genes were assigned to a putative function, all but three genes showed significant sequence similarity to gene sequences, of either known or unknown function, in the NCBI database (Table 5). No paralogs were identified in this genome.

**Table 3:** Nucleotide content and gene count levels of the genome

**Attribute**	**Value**	**% of total^a^**
Size (bp)	38,682	100
G+C content (bp)	18,998	49.11
Coding region (bp)	37,543	97.1
Total genes	40	100
RNA genes	0	0
Protein-coding genes	40	100
Genes in paralog clusters	0	0
Genes assigned to COGs	12	30
1 or more conserved domains		
2 or more conserved domains		
3 or more conserved domains		
4 or more conserved domains		
Genes with signal peptides	6	15
Genes with transmembrane helices	2	5
Paralogous groups	0	0

a) The total is based on either the size of the genome in base pairs or the total number of protein coding genes in the annotated genome.

**Table 5:** Vibriophage VvAW1 gene annotations*.

**Gene**	**Strand**	**Function or Similarity**	**Evidence or Organism**	**Accession Num.**	**% Id**	**E-Value**
1	-	DNA methylase	COG4646	COG4646	24	8.0e^-65^
2	-	hypothetical protein	Thalassomonas phage Ba3	NC_009990	43	2.0e^-63^
3	-	phage DNA transfer protein	*Xyella fastidiosa* M12	CP000941	35	1.0e^-19^
4	-	hypothetical phage protein	PHA00672	cl10253	nr	2.4e^-42^
5	-	hypothetical protein	none	n/a	n/a	n/a
6	-	phage tail collar protein	pfam07484	n/a	nr	1.5e^-05^
7	-	phage stabilization prot., gp 10	Xyella phage Xfas53	GQ421471	36	1.0e^-25^
8	-	hypothetical protein	Thalassomonas phage Ba3	NC_009990	34	2.0e^-25^
9	-	hypothetical protein	Thalassomonas phage Ba3	NC_009990	52	4.0e^-06^
10	-	P22 coat protein, gp5	pfam11651	cl0373	nr	1.3e^-82^
11	-	scaffold protein	EBPR podovirus	AE170876	36	4.0e^-30^
12	-	portal protein	EBPR podovirus	AE170876	38	1.0e^-127^
13	-	terminase large subunit	Shigella phage Shfl1	NC_015456	39	0.0e^+00^
14	-	terminase, small subunit	pfam03592	cl01513	nr	4.0e^-09^
15	-	hypothetical phage protein	PHA000821	cl10282	nr	5.8e^-10^
16	-	holin protein	Psychrobacter phage psymv2	AEO01029	46	1.0e^-13^
17	-	hypothetical protein	Acinetobacter phage AB1	ADO14413	48	4.0e^-21^
18	-	hypothetical protein	*Vibrio sp. AND4*	ZP_02196938	32	3.0e^-12^
19	-	endolysin	Bacteriophage P27	103807.1	37	2.0e^-11^
20	-	hypothetical protein	none	n/a	n/a	n/a
21	-	ssDNA binding protein	COG0629	COG0629	43	4.0e^-36^
22	-	hypothetical protein	none	n/a	n/a	n/a
23	-	con. protein, unknown func.	DUF2303	cl02338	nr	5.1e^-86^
24	-	hypothetical protein	CPS-53 prophage	AEQ15784	47	3.0e^-27^
25	-	hypothetical protein	Pseudomonas phage D3	NP_061574	34	3.0e^-19^
26	-	RNA-binding regulator protein	COG1551	COG1551	43	6.0e^-10^
27	-	exonuclease	pfam00929	cl0219	nr	1.1e^-17^
28	-	hypothetical protein	Pseudomonas phage F116	AY625898	40	0.0e^+00^
29	-	hypothetical protein	Pseudomonas phage F116	AY625898	43	0.0e^+00^
30	-	hypothetical protein	Pectobacteria phage ZF40	AFC22460.1	35	4.0e^-05^
31	-	hypothetical protein	none	n/a	n/a	n/a
32	+	lambda-like repressor, HTH mot.	SSF47413	IPR010982	nr	1.6e^-07^
33	+	hypothetical protein	Thalassomonas phage Ba3	NC_009990	46	3.0e^-19^
34	+	con. protein, unknown func.	DUF3268	cl13172	nr	1.7e^-29^
35	+	hypothetical protein	*Vibrio cholerae* MZO-3	ZP_01955042	40	1.0e^-47^
36	+	hypothetical protein	*Opitutacecae sp.* TAV5	ZP_09596846	50	1.0e^-27^
37	+	con. protein, unknown func.	DUF1367	cl06231	nr	1.7e^-51^
38	+	con. protein, unknown func.	DUF1364	cl06229	nr	5.1e^-38^
39	-	hypothetical protein	*Roseobacter denitrificans*	YP_771820	29	4.0e^-09^
40	-	cellulosome enzyme	*Acinetobacter baumanni*	ZP_05829744	36	1.0e^-92^

*Genes are listed by number, along with their predicted function, if known, followed by the nature of the evidence (e.g., COG group) that supports the functional classification. Genes with no functional prediction, but with significant (E < 10^-5^) sequence similarity to genes in the NCBI database as determined by blastp, are listed, followed by the name of the organism in which the similar gene was found.

**Table 4:** Number of genes associated with the 25 general COG functional categories

**Code**	**Value**	**%age**^a^	**Description**
J	0	0	Translation
A	0	0	RNA processing and modification
K	2	5	Transcription
L	3	7.5	Replication, recombination and repair
B	0	0	Chromatin structure and dynamics
D	0	0	Cell cycle control, mitosis and meiosis
Y	0	0	Nuclear structure
V	0	0	Defense mechanisms
T	2	5	Signal transduction mechanisms
M	1	2.5	Cell wall/membrane biogenesis
N	1	2.5	Cell motility
Z	0	0	Cytoskeleton
W	0	0	Extracellular structures
U	0	0	Intracellular trafficking and secretion
O	0	0	Posttranslational modification, protein turnover, chaperones
C	1	2.5	Energy production and conversion
G	0	0	Carbohydrate transport and metabolism
E	0	0	Amino acid transport and metabolism
F	0	0	Nucleotide transport and metabolism
H	0	0	Coenzyme transport and metabolism
I	0	0	Lipid transport and metabolism
P	0	0	Inorganic ion transport and metabolism
Q	0	0	Secondary metabolites biosynthesis, transport and catabolism
R	1	2.5	General function prediction only
S	3	7.5	Function unknown
-	28	70	Not in COGs

a) The total is based on the total number of protein coding genes in the annotated genome.

## Insights from the genome sequence

### Comparative genomics

**Figure 4:**
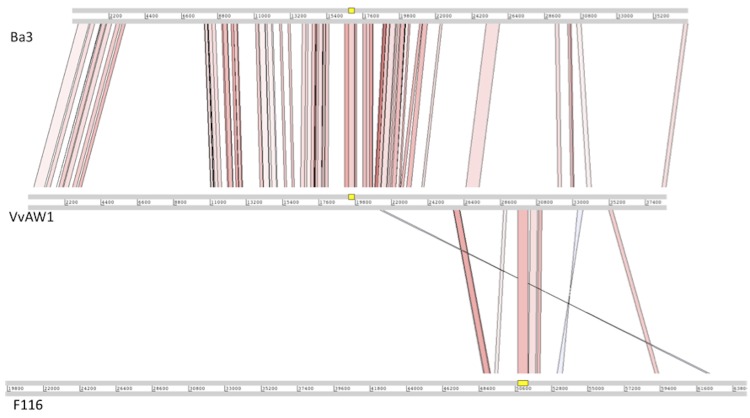
Whole genome comparison of Vibrio phage VvAW1 to Thalassomonas phage Ba3, and Pseudomonas phage F116. This figure was generated using the Artemis Comparison Tool (ACT) [21]. Genomes were aligned using WebACT, using default tblastx settings, with E-value set to 10e^-4^, and genetic code set to eubacterial. ACT display was set to show homologous regions with BLAST scores >40, and sequence similarity >25%.

Significant similarity was observed between Vibriophage VvAW1 and Ba3, which infects the coral pathogen *Thalassomonas loyana,* both in terms of gene order and gene homology (Figures 3 and 4). Bacteriophage genomes have been described as mosaic, with areas of intense similarity amalgamated with areas that appear to be unrelated [23]. The genome of VvAW1 displays extensive mosaicism, with some regions closely related to Ba3, and some apparently unrelated. Many of the areas of the genome that do not show homology to Ba3, show significant similarity to another phage, Pseudomonas phage F116 (Figure 4). Only one of the predicted VvAW1genes (gene 13) showed significant sequence similarity to both Ba3 and F116. Mosaic patterns in bacteriophage genomes support the theory that horizontal gene transfer plays a role in phage evolution [23].

## VvAW1 replication strategy

**Figure 5:**
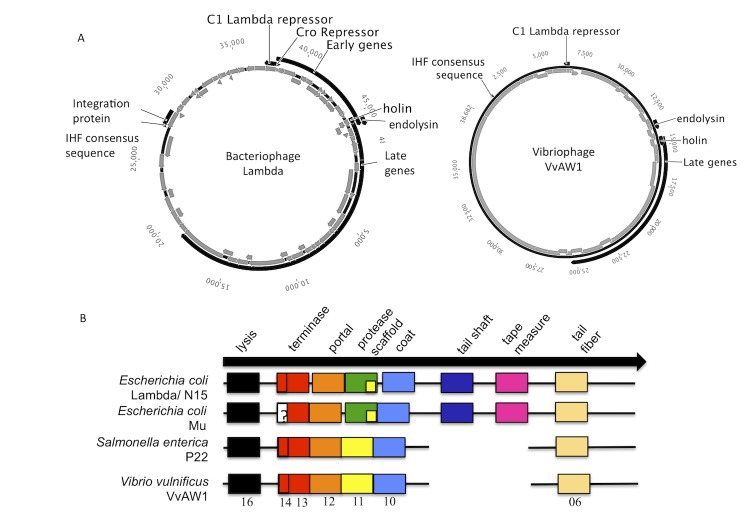
Comparison of similar regions of the lambda, and Vibriophage VvAW1 genomes. (A) Circularized genomes of lambda and VvAW1 highlighting synteny between the two phages. The genome of phage lambda was accessed from GenBank, accession number NC_001416 [25]. The genome maps were drawn using the Geneious software package [14].(B) Late genes of temperate phages. This figure was redrawn and modified from Casjens, 2003 [26]. Similar colors represent genes with conserved order, but not necessarily sequence similarity. Spaces between the colored squares indicate that additional genes lie between the indicated genes. The numbers below the VvAW1 genome indicate gene number as described in Table 5.

The life cycle and replication strategy of Vibrio phage VvAW1 have not been determined, however while propagating the phage it was found that infected cultures did not completely clear, and plaques were turbid (data not shown), suggesting that the bacteriophage is temperate [24]. Analysis of the genome sequence further supports the hypothesis that the phage is temperate. As determined by homology searches using the CDD, Pfam motif analysis, and InterProScan, the predicted protein product of gene 32 is a transcriptional regulator with homology to the Enterobacteria phage lambda (lambda) repressor C1, which is responsible for maintaining lysogeny in *E. coli*. The VvAW1 C1 homolog also displays a helix-turn-helix motif. The putative C1 repressor gene is a location of transcriptional divergence in the VvAW1 genome, similar to lambda (Figure 5a). The temperate bacteriophage lambda has a central regulatory circuit that has been well-studied. Divergently transcribed repressors (C1 and Cro) regulate passage into the lytic or lysogenic cycle [27]. Although sequence homology was not seen in gene product 31 to the Cro repressor, genome arrangement between the two phages is conserved (Figure 5a). The intergenic space between the C1 and Cro genes in lambda is the site of two key promoters involved in regulatory events. Although the promoters were not identified in this region of the VvAW1 genome, GC content in the intergenic spacer is low (37%) relative to the VvAW1 genome.

Synteny between VvAW1 and lambda persists in other regions of the genome. The lytic pathway of lambda, up-regulated by Cro production, leads to the transcription of “early” and “late” phage genes. These genes are located downstream of the Cro repressor. The early genes, which encode proteins involved in DNA replication, are transcribed first, followed by the morphogenetic or late genes, which encode phage assembly proteins. This modular organization of the genome is typical of tailed bacteriophages [28]. Temperate phages show a striking conservation of gene order with regard to their morphogenetic genes with very few exceptions to the clustering and specific order of these genes [26]. The genome of VvAW1 shows the gene clustering of function and conservation of gene order of early and late genes that is characteristic of temperate phages (Figure 5b). Notably, the VvAW1 genome is missing the “tail shaft” and “tape measure” genes, as is the case for the genome of the Salmonella phage P22. The absence of these genes in P22 can be attributed to the fact that P22 is a podophage, and therefore has a short tail. The absence of these genes in VvAW1 as well, corroborates the morphological and genomic evidence and further supports the inclusion of this phage in the family *Podoviridae*.

We were unable to identify an integrase gene in the genome of VvAW1. Integrase genes regulate the integration of viral genomes into the genome of their host, and in lambda this gene is located downstream of the C1 lambda repressor (Figure 5A) [25,29]. The integrase gene may be present and not sufficiently similar to other integrases to be identified by sequence similarity. It is also possible that VvAW1 replicates as a plasmid, which has been observed in F116, as well as Vibriophage VHS1 [30,31]. Immediately downstream of the intergrase gene in the lambda genome is the attP site, which contains integration host factor (IHF) binding sites. We have identified a region in VvAW1 that has the IHF binding consensus sequence AWWTCAANNNNTR downstream of the putative lambda-like repressor [32]. The consensus sequence lies within gene 40 in VvAW1. Gene 40 does not show homology to other integrase genes. Blastp analysis of gene 40 indicated homology to the dockerin type I cellulosome protein of several bacterial species. If the identified IHF sequence is part of the attP site of VvAW1, gene 40 could be of bacterial origin, as a result of genetic recombination.

## Conclusion

According to our analysis of the Vibriophage VvAW1 genome, this phage is most likely a member of the viral family *Podoviridae*. The genome shows modular organization and mosaicism. Portions of the genome show synteny with the genome of bacteriophage lambda. High sequence similarity was observed between VvAW1 and the Thalassomonas phage Ba3, as well as the Pseudomonas phage F116. Functional predictions of VvAW1 genes indicate the possibility of a lysogenic replication strategy, however an integrase gene could not be identified in the genome. It is possible that VvAW1 lysogenizes its host, without integrating into the host genome, replicating as a plasmid.
